# Treatment of Hypertrophic Scar in Human with Autologous Transplantation of Cultured Keratinocytes and Fibroblasts along with Fibrin Glue

**DOI:** 10.22074/cellj.2015.511

**Published:** 2015-04-08

**Authors:** Ehsan Taghiabadi, Parvaneh Mohammadi, Nasser Aghdami, Nasrin Falah, Zahra Orouji, Abdoreza Nazari, Saeed Shafieyan

**Affiliations:** Department of Stem Cells and Developmental Biology at Cell Science Research Center, Royan Institute for Stem Cell Biology and Technology, ACECR, Tehran, Iran

**Keywords:** Hypertrophic Scar, Fibrin Glue, Fibroblast, Keratinocyte, Scaffold

## Abstract

**Objective:**

Hypertrophic scar involves excessive amounts of collagen in dermal layer and may be painful. Nowadays, we can’t be sure about effectiveness of procedure for hypertrophic scar management. The application of stem cells with natural scaffold has been the best option for treatment of burn wounds and skin defect, in recent decades. Fibrin glue (FG) was among the first of the natural biomaterials applied to enhance skin deformity in burn patients. This study aimed to identify an efficient, minimally invasive and economical transplantation procedure using novel FG from human cord blood for treatment of hypertrophic scar and regulation collagen synthesis.

**Materials and Methods:**

In this case series study, eight patients were selected with hypertrophic scar due to full-thickness burns. Human keratinocytes and fibroblasts derived from adult skin donors were isolated and cultured. They were tested for the expression of cytokeratin 14 and vimentin using immunocytochemistry. FG was prepared from pooled cord blood. Hypertrophic scars were extensively excised then grafted by simply placing the sheet of FG containing autologous fibroblast and keratinocytes. Histological analyses were performed using Hematoxylin and eosin (H&E) and Masson’s Trichrome (MT) staining of the biopsies after 8 weeks.

**Results:**

Cultured keratinocytes showed a high level of cytokeratin 14 expression and also fibroblasts showed a high level of vimentin. Histological analyses of skin biopsies after 8 weeks of transplantation revealed re-epithelialization with reduction of hypertrophic scars in 2 patients.

**Conclusion:**

These results suggest may be the use of FG from cord blood, which is not more efficient than previous biological transporters and increasing hypertrophic scar relapse, but could lead to decrease pain rate.

## Introduction

Hypertrophic scarring is the main negative outcome following deep partial-thickness wounds and burns. These scars are unsightly, deforming and may reduce, but never totally disappear. Tissue engineering is the principal therapeutic strategy for treatment of post-burn hypertrophic scars. Deformed skin is replaced by scaffolds and/or cells (1). In tissue engineering the healing valence of the patient is enhanced and guided, enabling damaged skin to be reformed and its natural functions repaired (2). In tissue engineering, one of the fundamental purposes of cell biologists is to stimulate cell proliferation by tailored culturing conditions (3,4). The ideal scaffold should have tissue mechanical traits, show immunologic integrity, support cell adhesion, proliferation and migration. One of the natural biodegradable and biocompatible scaffold is fibrin glue, which provides an excellent bed for cell attachment, migration, proliferation and wound healing (5). Fibrin glue ( FG ) made up of two components fibrinogen and thrombin that are utilized to glue them together. Thrombin is the same of enzyme changes fibrinogen three-dimensional gel. FG plays a main role in homeostasis, inflammation, wound healing and angiogenesis regulation (6). Degradation of FG scaffold induces acute phase response in wound healing. Collagen synthesis organization and fibril adjustment of different cell types is increased in fibrin scaffolds. In contact with FG, cells will slowly replace the FG scaffold by tissue-specific extracellular matrix. Combining different skin cell types cause selforganization within the scaffold, mimicking the natural dermis. Migration and proliferation of fibroblasts in the fibrin scaffold in 2-3 days has been seen in co-cultures of keratinocytes with dermal fibroblasts (7). Fibroblasts were stimulated to generate collagen in response to transforming growth factor beta ( TGF-β ) in a fibrin scaffold (8,9) so; these properties are the main reasons of FG application in skin tissue engineering. The commercial FG made-up of lyophilized pooled human fibrinogen and thrombin. These structures may increase a risk of an immunological reaction and disease transfer. The more difficult to cost of autologous fibrin glues are often incorporated into a preexisting blood bank. Therefore, low cost, short preparation time, and eschew the potential risk of foreign body reactions all play a role of application cord blood fibrin for skin tissue engineering (6). In this study, we transplanted a composite of autologous fibroblasts and keratinocytes into excised burn hypertrophic scars in 8 patients using cord blood FG and monitored clinical outcomes for 8 weeks. We tested whether the effect of fibrin gel enriched with human autologous fibroblasts and keratinocytes has beneficial effects on the final treatment outcome in 2 patients with excised burn hypertrophic scars. 

## Materials and Methods

### Patient selection and method of hypertrophic excision

In this case series study, 8 patients selected with hypertrophic scar due to full-thickness burns. The size of scars was 10 cm^2^. Five cases were female ( 20-45 years ) and 3 were male ( 25-30 years ). The average distribution age was 29 years. The punch 3 cm^2^of hypertrophic scar were punched and completely removed by scalpel and then grafted by FG sheet that follow up 8 weeks. 

### Primary human keratinocyte and fibroblast cell culture

This study was approved by Ethical Commitee of Royan Institute. After obtaining informed consent, normal human keratinocyte and fibroblast cells were derived from 8 patients with hypertrophic scar. Skin biopsies were obtained from the buttock for keratinocytes and the ear for fibroblasts. All donors had serologically negative human immunodeficiency virus, hepatitis virus type B, hepatitis virus type C and syphilis. Each biopsy was washed in Hank’s buffered salt solution ( HBSS, Gibco, USA ) containing 100 U/ml penicillin and 100 µg/ml streptomycin ( Gibco, USA ) and was incubated in dispase ( Gibco, USA, 1.2 U/ml ) solution at 4˚C overnight. After separaC tion of the epidermis from the dermis, the epidermis and dermis were cut into small pieces and then keratinocyte and fibroblasts were isolated from the epidermis and dermis by incubation in 0.25% Trypsin/Ethylenediaminetetraacetic acid ( EDTA ) ( Gibco, USA ) for 10 minutes at 37˚C and 0.1% collagenase I ( Sigma, USA ) enzyme for 4 hours at 37˚C. Trypsin/EDTA and collagenase I enzymes were neutralized with culture medium containing 10% serum and a cell suspension obtained by pipetting. The supernatant was discarded and the cell suspension was filtered using a 70 μm nylon mesh filter ( BD, USA ). Each cell suspension was centrifuged for 5 minutes at 1500 rpm and the cell pellet re-suspended in normal saline to be counted using a hemocytometer ( Bright-Line™, USA ). Finally, isolated keratinocyte was cultured by seeding 6-well cell culture plate in fibroblast basal medium ( FBM, Gibco, USA ) + keratinocyte growth medium ( KGM, Gibco, USA ) at 37˚C in 5% CO_2_at 95% humidity. Isolated dermis fibroblasts were cultured in DMEM-F12 ( Gibco, USA ) containing fetal bovine serum ( FBS, 10%, Hyclone, USA ), L-glutamine ( Gibco, USA ), 100 U/ml penicillin and 100 µg/ml streptomycin at 37˚C, 5% CO_2_at 95% humidity. The culture medium was changed every two days. A final density of 2×10^6^cells/ml dermis fibroblasts and 5×10^6^cells/ml epidermis keratinocytes were used at third passage and first passage for culture on FG, respectively. The result of microbial endotoxin and mycoplasma tests in cultivated keratinocytes and fibroblasts should be negative if they are to be used for transplantation. 

### Preparation of a composite of fibrin with fibroblast and keratinocyte cells

**Fig.1:**
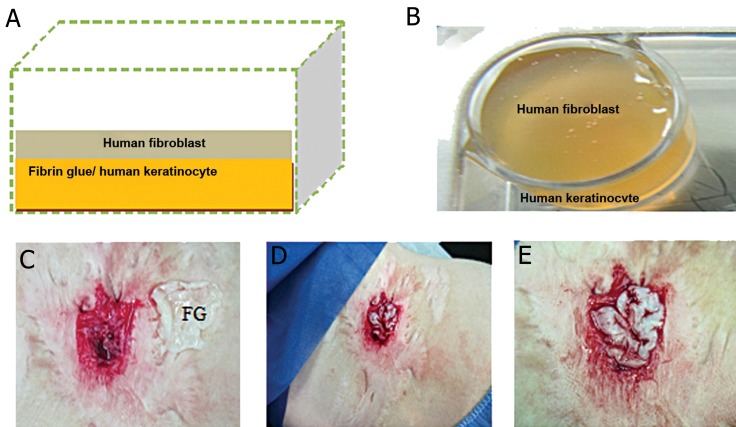
A. Schematic presentation of the fibrin glue (FG) scaffold: cultured human dermal fibroblast obtained on fibrin glue containing human keratinocyte cells, B. This mixed thrombin-keratinocyte part was put together with the fibrin part on the floor of the culture plate, then fibroblast cells was seeded on surface of fibrin glue, C. The excision of hypertrophic burn scar, D and E. Transplantation of fibrin glue contains autologous keratinocytes and fibroblast.

Human FG derived from pooled cord blood was obtained from the Royan Regenerative Medicine Center for medical application and was used in this study. The FG was created as follows: about 5×10^6^autologous human keratinocytes were mixed with 3 ml of thrombin component ( 500 IU/ml ). The mixed "cell thrombin part" was added to 3 ml fibrinogen ( 40 mg/ml ) on the floor of 60 mm cell culture dishes. After clot formation of FG ( 5 minutes ), 2×10^6^autologous fibroblasts were seeded on solidification of the FG. This scaffold was incubated at 37˚C in 90% humidity and 5% CO_2_-concentration for 3 hours in FBM (Fig.1A,B). FG containing autologous fibroblast and keratinocyte cells were removed from the 60 mm cell culture dishes with the aid of two forceps and transplanted upside down on the excised hypertrophic scars (Fig.1C,D,E). 

### Immunohistochemical (IHC) analysis for characterization of cultured cells

Human keratinocytes and fibroblasts were fixed for IHC staining using 4% paraformadehyde ( Sigma, USA ) for 5 minutes, then washed 2 times. Each time for 5 minutes using Dulbecco’s phosphate buffered saline [PBS/Tween ( PBST ); 0.05%, Sigma, USA]. The permeability of the cells were determined by adding Triton X-100 ( MERK, Germany, 0.2% ) and incubated for 10 minutes at room temperature and then washed twice with PBS/Tween 0.05%, each time for 5 minutes. Cells were blocked by blocking buffer containing 10% goat serum ( Sigma, USA ) and then incubated for 20 minutes at 37˚C, to block non-spei cific antibody binding, then washed twice for 5 minutes. Primary antibodies including vimentin ( Sigma, USA ) and cytokeratin 14 ( Abcam , UK ) were added at 1/100 dilution and incubated for 2 hours at room temperature. The cells were washed twice by PBS/ Tween 0.05%, each time for 10 minutes and then fluorescein isothiocyanate ( FITC ) goat anti mouse ( Dako, USA ) secondary antibody was added at 1/200 dilution and incubated for 1 hour at room temperature. Finally, cells were washed by PBST and examined under a fluorescent microscope. Dapi dye was used for nuclear staining. 

### Flowcytometry for further characterization of cultured cells

Human dermal fibroblasts passage 3 were resuspended in a 3% bovine serum albumin ( BSA; Sigma-Aldrich, USA ) solution and incubated for 20 minutes at room temperature, following manufacturer-recommended concentrations, with mouse antihuman antibody vimentinPhycoerythrin ( Sigma, USA ). Cells were subsequently washed with phosphate-buffered saline ( PBS; Gibco, USA ), centrifuged and analyzed on a FACs Calibur Flow Cytometer ( BD Biosciences, USA ) using the CELL Quest software version 3.3. 

### Gene expression analysis by reverse transcriptase polymerase chain reaction (RT-PCR)

Gene expression for fibroblast extracellular matrix component ( type 1 collagen ) was evaluated by RTPCR technique. Expression level of the targeted gene was normalized to *GAPDH*. The following primers specific for *GAPDH* sequences were used for RTPCR: forward: 5΄ATGCCTGGTGAACGTGGT3΄, reverse: 5΄AGGAGAGCCATCAGCACCT3΄. Targeted primer was designed with primer 3. Total RNA was extracted from fibroblast cells with trayzol ( Sigma, USA ). Extracted RNA was treated with 1 U/ml of RNase-free DNase I ( EN0521, Fermentas, Germany ) per 1 mg of RNA in order to eliminate residual DNA in the presence of 40 U/mL of ribonuclease inhibitor ( E00311, Fermentas, Germany ) and 1X reaction buffer with MgCl_2_( Sigma, USA ) for 30 minutes at 37˚C. To inactivate DNase I, 1 ml of 25 mM EDTA ( Sigma, USA ) was added and incubated at 65˚C for 10 minutes. Standard reverse transcriptase ( RT ) reactions were performed with 2 µg total RNA using oligo ( dt ) ( Fermentas, Germany ) as a primer and a Revert Aid TM First Strand cDNA Synthesis Kit ( K1622, Fermentas, Germany ) based on the manufacturer’s instructions. For every reaction set, one RNA sample was prepared without Revert Aid TMM MuLV RTreaction to provide a negative control in the subsequent PCR. To minimize variation in the RT reaction, all RNA samples from a single experimental setup were reverse transcribed simultaneously. Reaction mixtures for PCR included 2 mL cDNA, 1X PCR buffer ( AMSTM, CinnaGen Co., Tehran, Iran ), 200 mM dNTPs, 0.5 mM of each antisense and sense primers ( AMSTM, CinnaGen Co., Tehran, Iran ), and 1U Taq DNA polymerase ( AMSTM, CinnaGen Co., Tehran, Iran ). The accession number of primer is NM-000088.3 and length of ladder is 50 base pairs ( bp ). The following primers specificly for human collagen type I chain sequences were used for RT-PCR: forward: 5΄TTGCCGACAGGATGGAGAAGGA3΄, reverse: 5΄AGGTGGACAGCGAGGCCAGGAT3΄. 

### Histological assessment

At the end of the 8-week study period, two biopsies were harvested from the wound area of patients, and normal skin fixed in 10% buffered formalin ( Sigma, USA ) for 24 hours. Then cut into five to seven 5 µm sections, prepared for Hematoxylin and eosin ( H&E ) and Masson’s Trichrome ( MT ) staining. The histological analysis using conventional microscopy with an Olympus BX61. Digital Images were captured by using an Olympus DP70, 12 megapixel camera ( Olympus, USA ). 

### Epidermal thickness measurement

Epidermal thickness of the formed neoskin was assessed from H&E stained histological sections of both treatment and normal skin after 2 months. Five tissue sections for each patient were randomly evaluated selecting 10 high-power fields in each section and performing 10 measurements of the epidermal thickness in the fields. 

### Analysis

Image analysis for the quantification of epidermal maturation was performed using Image J image analysis software ( Wayne, Rasband, NIH, USA ). Data analysis of epithelial maturation and dermal differentiation was carried out by one-way analysis of variance ( ANOVA ) with Turkey’s post tests ( GraphPad Prism 4.02 ). Values of p less than 0.05 were considered significant. All data were reported as mean ± standard deviation ( SD; n=10 ). 

## Results

### Cell culture and characterization and delivery in a fibrin glue

**Fig.2:**
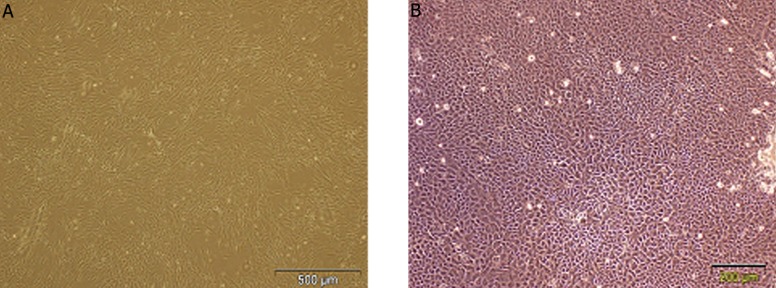
Morphologic appearance of human dermal fibroblast and keratinocyte cultured cells and their exit from fibrin. A. Appearance of cultured fibroblast cells on tissue culture plastic by day 6 after seeding and B. Appearance of cultured keratinocyte cells on tissue culture plastic by day 10 after seeding.

**Fig.3:**
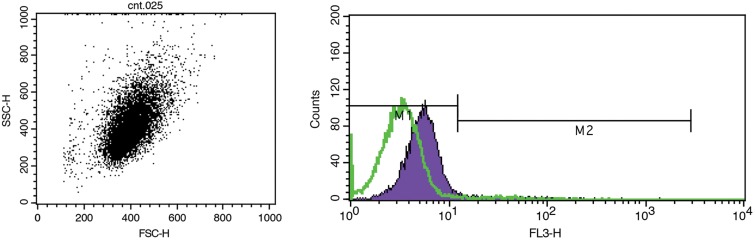
Shows the dot plot and histogram corresponding to the analysis of cell suspension from human dermal fibroblast cells. Characterization of the fibroblast cells for cytoplasm marker: vimentin. The bars on the peak levels of histogram M1 (Isotope control) and M2 are a measure of positive expression vimentin.

**Fig.4:**
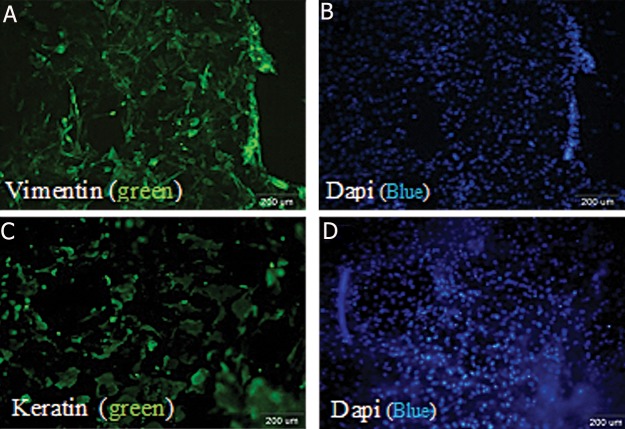
Immunocytochemistry of cultured fibroblast and keratinocyte cells for vimentin and keratin 14 (K14), respectively. Fibroblast and keratinocyte cultured cells were grown on 4 wells culture plate and immunostained with vimentin and K14 markers. These representative examples illustrate that the cultured cells were positive for fibroblast and keratinocyte markers (A. Vimentin and C. K14). Fluorescent compound, vimentin and K14 (green) and Dapi (Blue) (B and D) was used to stain cells and nucleus.

No adverse incidents happened when taking the skin biopsies from patients or during the isolation of fibroblasts and keratinocytes using an enzymatic procedure and subsequent cell culture. The morphology of cultivated fibroblasts and keratinocytes is displayed in (Fig.2A,B). By approximately 3 days, fibroblast flasks contained spindle-shaped cells. Autologous fibroblasts for utilization in patients were applied within the first 3 passage. But autologous keratinocyte cells were harvested in primary passage. After 6 days, keratinocytes flasks contained polygonal-shaped cells. Under a good cultivation procedure fibroblasts and keratinocytes became confluent within 6 and 10 days, respectively. The flowcytometry profile of fibroblast cells illustrated the following specifications and percentage positivity of cytoplasm marker: vimentin ( 98 % ) (Fig.3). This corresponded well with immunostaining of cells plated on a 6 wells culture plate demonstrated expression of vimentin (Fig.4A,B). The result of immunostaining keratinocytes cultivated on a 4 wells culture plate demonstrated expression of K14 (Fig.4C,D). 

### RT-PCR displayed expression of type I collagen mRNA transcripts in cultivated dermal fibroblasts

**Fig.5:**
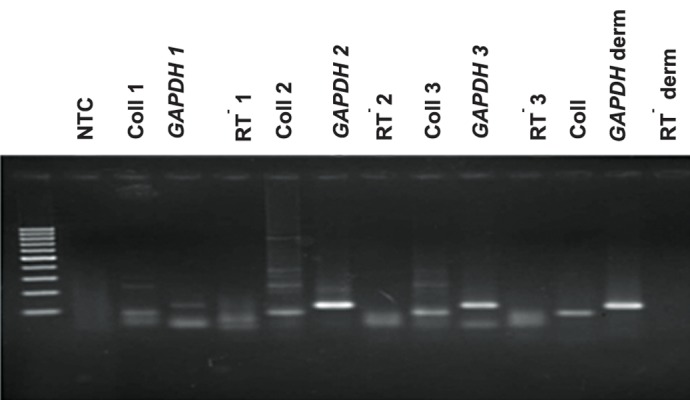
For different passage of human dermal fibroblast cells passages 1 (Coll 1), 2 (Coll 2), 3 (Coll 3), the analysis of the expression profiles for collagen I RNAs showed no difference as compared with normal human skin tissue. NTC: No template control; Coll 1: Collagen (sample 1); RT: Negative control and GAPDH: Glyceraldehyde 3-phosphate dehydrogenase.

To characterize the level of type I collagen mRNA in cultivated human dermal passage 1, 2 and 3 and human normal skin, RT-PCR was accomplished with total RNA derived from cultivation of dermal fibroblast 3 patients for each passage. Scanning densitometry of the RT-PCR product, after correction by the value from the internal standard, showed no difference in the expression of type I collagen mRNA transcripts in fibroblast cultures passage 1, 2, 3 from patients, as compared with human normal skin tissue (Fig.5). 

### Application of FG containing fibroblast and keratinocyte cells to human hypertrophic scar

We tested delivery of autologous fibroblast and keratinocytes with a FG to hypertrophic scar wounds ( n=8 ). Skin biopsy of patients isolated approximately 4 weeks before the transplantation to allow for appropriate *in vitro* development of keratinocyte and fibroblast cultures by the day of surgery. 

The total FG content ( fibrinogen and an equal volume of thrombin ) was approximately 6 mL in cell culture 60 mm^2^. These hypertrophic scar wounds contained approximately 9×10^5^keratinocytes/cm^2^and 3.5×10^6^fibroblasts. Healing of the hypertrophic scar occurred following 1 week after transplantation and by no later than week 8 (Fig.1A,B). 

### Epidermal morphogenesis is modulated by FG and fibroblast and keratinocyte cells

**Fig.6:**
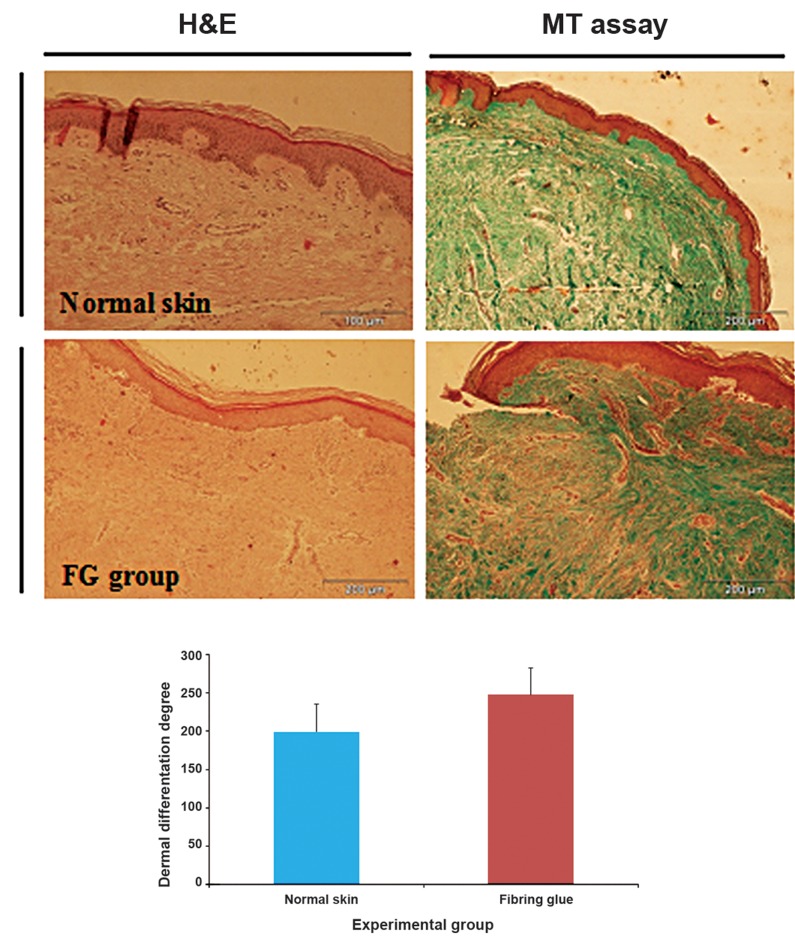
A. Hematoxylin and eosin (H&E) staining of normal skin (control), B. Histological analysis of the normal skin (control), stained with Masson Trichrome (MT), C. Composite of fibrin glue with fibroblast and keratinocyte cells, groups stained with H&E after 2 months evidencing the differences in the thickness of the epidermis among the groups (neodermis quality evaluation), D. Histological analysis of FG groups, stained with MT, evidencing in higher magnification pictures the differences in the dermal matrix organization among the groups, MT assay results showed of the dermal degree of differentiation obtained after a graded qualitative and E. Comparison of MT assay in control and fibrin glue groups showed no statistical difference (p>0.05), Data are shown as mean ± standard deviation (SD), n=10.

Re-epithelization is a basic success in hypertrophic wound healing. Therefore, morphogenesis and quality of the epidermis formed in test group was compared with normal skin. From the H&E images, it was clear that the newly formed epidermis in implantation area is not different from control group (Fig.6A,B). Epidermis of the FG group was not significantly thicker than the epidermis of control groups (Fig.6B). The implanted FG containing autologous fibroblast and kertinocytes also seemed to affect the degree of dermal maturation (Fig.6C,D). However the FG group appeared to have a higher degree of differentiation than the dermal tissue formed in the control condition (Fig.6D). 

### Application of autologous fibroblast and keratinocyte cells to human hypertrophic scar wounds 

**Fig.7:**
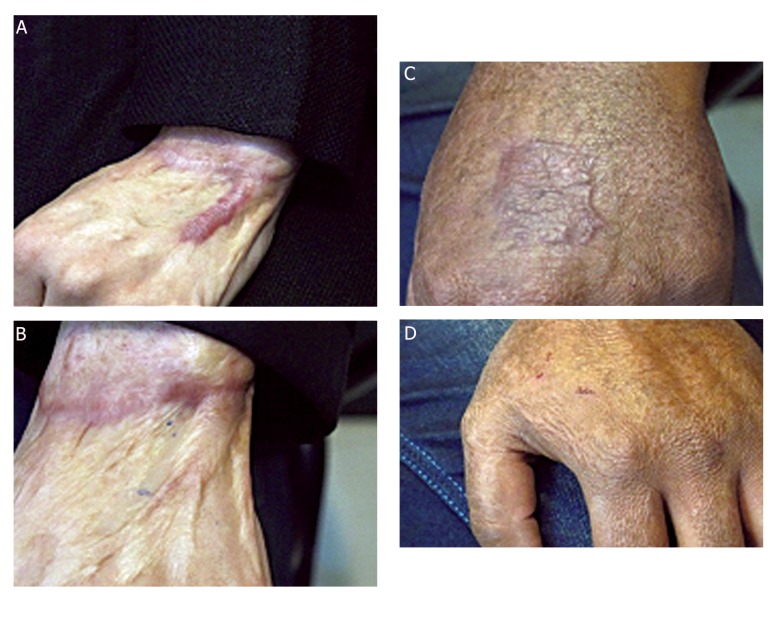
Application of autologous and keratinocyte cells with fibrin glue to human hypertrophic scar wounds. A. Hypertrophic scar over the hand before treatment, B. Reduction of hypertrophic scar area after 2 months. The wound bed has filled in completely, C. The hypertrophic scar wound over hand before treatment and D. Application of autologous fibroblast and keratinocyte cultured cells in fibrin glue to the now healing wound. After 2 months, the wound is almost healed.

Eight patients had hypertrophic scar wounds on the forearm, chest, leg and hand. In each of these 6 subjects, the hypertrophic scar did not significantly change in size after 8 weeks in comparison with treatment by FG (Fig.7A,B). But two patients that had hypertrophic scars on their hands, showed excellent epithelization and reduction in their wound size after 8 weeks (Fig.7C,D). All wounds were completely closed in three weeks after the surgery. No adverse events were noted. Follow-up of patients continued for 8 weeks. This finding suggests that in hypertrophic scar which generally heal uneventfully, autologous fibroblasts and keratinocytes in cord blood FG application may lead to more accelerated resurfacing. 

## Discussion

Hypertrophic scar formation happens because of abnormal wound healing and plentiful amounts of dermal collagen after burn skin injury (10). Some researchers have focused on the immune regulation of collagen generation and deposition. Different kinds of cytokines have been shown to adjust collagen deposition, including, platelet-derived growth factor ( PDGF ) and TGF-β. Unusual expression of these factors or genes may cause excessive deposition of the dermal matrix in hypertrophic scars (11). There is no ideal therapy for hypertrophic scars (12). Therapeutic methods include different combinations of triamcinolone, surgical excision, pressure therapy, silicone, radiotherapy. Surgical excision for keloids and hypertrophic scars has a relapse rate ranging from 45-100%. Utilizing an autograft for covering the excision site decreases the risk of forming hypertrophic scars at the donor site. There have been some experiments using an artificial neodermis. Integra was applied to enhance accelerate wound after excision of a large hypertrophic scar in addition to 5% imiquimod cream. There was no recurrence mentioned after 8 month follow-up (12). In our study we used cord blood FG containing fibroblast and keratinocyte cells for coverage of the hypertrophic scar excision site. One of the hypotheses in the pathogenesis of hypertrophic scar is that enhanced mechanical pressure located on the healing wound misaligns the orientation of the collagen formation and caused hypertrophic scar formation (13). In fact, during the healing of a skin wound, the defect is provisionally closed with fibrin clot. This clot is afterward permeated by inflammatory cells, fibroblasts, keratinocyte and granulation tissue (14). We think that FG scaffold is an acutely useful method in surgery, graft adherence and cellular migration. Also, it is an effective carrier system for cultivated keratinocytes, fibroblasts and growth factors. Currie et al. (5) has evaluated the positive and negative role of FG in skin tissue engineering. One of the major advantages of our new culture system is the use of widely available cord blood samples for the fabrication of fibrin gels. Our source of FG differs from a commercially available fibrin gel. The cord blood is a safe and available source for FG. Cultivation of keratinocytes on FG and fibroblasts is easy to handle. In hypertrophic scars, dermal fibroblasts represent production of collagen and matrix metalloproteinase leading to distinguished dermal collagen proliferation (15). Therefore, even after excision of the hypertrophic scar it will produce dermal collagen proliferation. However, it is our hypothesis that FG will discontinue the uncommon process of dermal collagen proliferation at the hypertrophic scar excision site (16). Our results from this small pilot study display that with the use of FG containing autologous fibroblasts and keratinocytes after hypertrophic scar excision, recurrence is not lower compared with historic data from other studies using commercial FG. Also in this study we are able to differentiate between relapse and treatment of except based on clinical appearance of the wound. In future studies, it may be beneficial to evaluate clinical appearance and histology of the scar tissue for long follow-up ( 12-24 months ). 

Additional future research is necessary to further evaluate this treatment option and to determine the effectiveness of using fibrin scaffold with anti scar drug and cell therapy after hypertrophic scar excision. 

## Conclusion

This study provides evidence that cord blood FG containing fibroblast and keratinocyte can be produced within a short time period. Implantation of the fibroblasts and keratinocytes into hypertrophic scar excisional wounds, led to prolonged dermal remodeling, and increased dermal degree of maturation. FG re-epithelization was characterized by the presence of ridge-like structures, and by a higher degree of epidermal maturation. Our further findings suggest that the transplanted FG, containing fibroblast and keratinocyte might be acting through paracrine effects of autologous cells. Thereby, they influence the different aspects of skin regeneration. The simplicity of the used methodology indicates short time period for FG production. There was not any notable difference between efficacy of cord blood FG and commercial FG. But the cord blood is a safe, cheap and available source for fabrication of FG. 
